# Evaluation of the percentage of ganglion cells in the ganglion cell layer of the rodent retina

**Published:** 2013-06-27

**Authors:** Cassandra L. Schlamp, Angela D. Montgomery, Caitlin E. Mac Nair, Claudia Schuart, Daniel J. Willmer, Robert W. Nickells

**Affiliations:** 1 Department of Ophthalmology and Visual Sciences, University of Wisconsin, Madison, WI; 2 Pathology and Laboratory Medicine, University of Wisconsin, Madison, WI; 3 University Eye Hospital, Otto-von-Guericke University, Magdeburg, Germany

## Abstract

**Purpose:**

Retinal ganglion cells comprise a percentage of the neurons actually residing in the ganglion cell layer (GCL) of the rodent retina. This estimate is useful to extrapolate ganglion cell loss in models of optic nerve disease, but the values reported in the literature are highly variable depending on the methods used to obtain them.

**Methods:**

We tested three retrograde labeling methods and two immunostaining methods to calculate ganglion cell number in the mouse retina (C57BL/6). Additionally, a double-stain retrograde staining method was used to label rats (Long-Evans). The number of total neurons was estimated using a nuclear stain and selecting for nuclei that met specific criteria. Cholinergic amacrine cells were identified using transgenic mice expressing Tomato fluorescent protein. Total neurons and total ganglion cell numbers were measured in microscopic fields of 10^4^ µm^2^ to determine the percentage of neurons comprising ganglion cells in each field.

**Results:**

Historical estimates of the percentage of ganglion cells in the mouse GCL range from 36.1% to 67.5% depending on the method used. Experimentally, retrograde labeling methods yielded a combined estimate of 50.3% in mice. A retrograde method also yielded a value of 50.21% for rat retinas. Immunolabeling estimates were higher at 64.8%. Immunolabeling may introduce overestimates, however, with non-specific labeling effects, or ectopic expression of antigens in neurons other than ganglion cells.

**Conclusions:**

Since immunolabeling methods may overestimate ganglion cell numbers, we conclude that 50%, which is consistently derived from retrograde labeling methods, is a reliable estimate of the ganglion cells in the neuronal population of the GCL.

## Introduction

Retinal ganglion cells comprise only a percentage of the neurons actually residing in the ganglion cell layer of the rodent retina. The estimate most often cited and used for calculations is 41.6%. This value was derived by Jeon and colleagues for the mouse retina (C57BL/6) [1] by estimating the total ganglion cell numbers using axon counts from cross sections of optic nerves, and total neurons in the ganglion cell layer (GCL) from ethidium-stained retinal whole mounts. A sample of publications using this estimate in the past 2 years can be found in these references [2–7]. Other methods for estimating ganglion cell numbers use either retrograde labeling protocols or staining of ganglion cell-specific markers, while the total neurons are typically estimated using a DNA or nucleoprotein stain. The method used to label ganglion cells can dramatically affect the estimate of the percentage of ganglion cells in the GCL, however, and estimates have been published ranging from 68% to 36% depending on the method. The percentage of ganglion cells, and the method used to derive this value, is potentially important. Many investigators use a count of total neurons in the GCL as the outcome metric for studies involving ganglion cell loss, to overcome technical problems such as early onset loss of ganglion cell gene expression [8, 9], retrograde dye toxicity [10], or compromise to axonal transport in pathologic conditions [11, 12]. Numbers of ganglion cells are then calculated using the estimate of ganglion cell percentage.

Ultimately, if corrections are used to present data in the literature as actual ganglion cell loss, there should be some standardization of what value is accurate. We set out to determine if different methods yield similar or different results for the percentage of ganglion cells in the GCL of the mouse retina.

## Methods

### Animals

Long-Evans rats (Sprague-Dawley, Madison, WI) and C57BL/6J mice (Jackson Laboratory, Bar Harbor, ME) were handled in accordance with the Association for Research in Vision and Ophthalmology Statement on the Use of Animals for Research. All methods were reviewed and approved by the RARC of the University of Wisconsin. Cholinergic amacrine cells in the ganglion cell layer were genetically labeled by crossing *Rosa26R-LoxP-tdTomato* reporter mice with a transgenic line expressing Cre recombinase under the control of the choline acetyltransferase (*Tg(Chat-cre)GM24Gsat*). These mice were a gift from Dr. Miles Epstein at the University of Wisconsin. For retrograde labeling experiments, rats and mice were anesthetized with ketamine (90 mg/kg) and xylazine (9 mg/kg).

### Retrograde labeling with combined FluoroGold and 4',6-diamidino-2-phenylindole

After the fur was shaved from the head, the overlying skin was treated with Betadine, and the skull was exposed with a midline incision. Two small holes were drilled 4–5 mm posterior to the bregma, one on either side of the midline suture to expose the brain. Two µl of 50:50 of 3% FluoroGold (Fluorochrome, Denver, CO) and 300 ng/ml 4’,6’-diamindino-2-phenylindole (DAPI; Thermo Fisher Scientific, Waltham, MA) in sterile phosphate buffered saline (PBS; 100 mM phosphate buffer, pH 7.2, 150 mM NaCl) were injected using a Hamilton syringe attached to a micromanipulator. The injection protocol entailed inserting the needle 1.5 mm into the brain (into the superior colliculus) and injecting 0.5 µl/min over the course of 4 min. Injections were given on both sides of the brain. The exposed scalp was closed using liquid sutures. Rats were injected using the same procedure, except that 8 µl of solution was injected over three locations in each side of the brain, through holes in the skull made at 7.5 mm behind the bregma and 2.0 mm on either side of the midline. Injections were made at a depth of 4.0 to 4.5 mm. Animals were analyzed at 4 days after the labeling protocol. Eyes were enucleated from euthanized animals after the superior quadrant was marked with an ophthalmic cautery. Whole eyes were fixed in 4% paraformaldehyde (PA) in PBS for 1 h at 22 °C, after which the retinas were removed, washed in PBS, and mounted on glass Plus slides (Thermo Fisher Scientific) with the GCL facing up. Relaxing cuts were made to create a whole mount with individual quadrants (superior, inferior, nasal, temporal) as the lobes of the whole mount. Additional retinas were processed for sectioning by embedding them in JB4-Plus glycol methacrylate (Polysciences, Warrington, PA) and sectioning at 1–2 µm.

### Retrograde labeling with 1,1'-dioctadecyl-3,3,3′3′-tetramethylindocarbocyanine perchlorate or FluoroGold

The brain was exposed as described, except that the superior colliculus was further exposed by aspirating the overlying cortex to a depth of 1 mm. Pledgets of gel-foam soaked in either ethanol containing 2% 1,1'-dioctadecyl-3,3,3′3′-tetramethylindocarbocyanine perchlorate (DiI; Anaspec, Freemont, CA) or PBS containing 2% FluoroGold were then packed into the space. Animals were euthanized 4 days later for evaluation. Whole mounts of retinas were made as described and counterstained with either DAPI (300 ng/ml) or TO-PRO-3 (1:3,000 dilution, Molecular Probes, Eugene, OR).

### Neuronal-specific nuclear protein and BRN3 immunostaining of whole mounts

Immunostaining of retinal whole mounts was performed essentially as described by Templeton et al. [13] for neuronal-specific nuclear protein (NeuN) and Nadal-Nicolás et al. [14] for BRN3, both with minor modifications. Enucleated eyes were fixed for 50 min at 22 °C in PBS containing 4% PA, washed in PBS, and then dissected to remove the anterior chamber and lens. For NeuN staining, eyecups were incubated in PBS containing 0.3% Triton-X100 and 10% horse serum for 3.5 h at 22 °C. Following a rinse in PBS, they were then incubated in PBS containing a 1:250 dilution of a rabbit polyclonal antibody against NeuN (MABN140, EMD Millipore, Billerica, MA) for 3 days at 4 °C. After washes in PBS, the eyecups were then incubated with a 1:1,000 dilution of goat anti-rabbit immunoglobulin G (IgG) conjugated to fluorescein isothiocyanate (111–095–003, Jackson ImmunoResearch Laboratories, West Grove, PA) for 2 days at 4 °C. After washing, the retinas were removed, whole mounted, and stained for 5 min with PBS containing 300 ng/mL DAPI. For BRN3 staining, dissected eye cups were incubated in PBS containing 0.5% Triton-X100 for 3.5 h at 22 °C. They were then transferred into PBS with 0.5% Triton-X100 and 2% donkey serum (Jackson ImmunoResearch) containing 1:50 dilutions of antibodies against BRN3A (mouse monoclonal MAB1585, EMD Millipore) and BRN3B (goat polyclonal cs-31989, Santa Cruz Biotechnology, Dallas, TX) overnight at 22 °C. After incubation, the eyecups were then washed in PBS with 0.5% Triton-X100 and 2% donkey serum containing 1:500 dilutions each of donkey anti-mouse IgG conjugated to Alexa 594 (Molecular Probes) and donkey anti-goat IgG conjugated to fluorescein isothiocyanate (Jackson ImmunoResearch) for 2 h at 22 °C. After washing, the retinas were removed, whole mounted, and stained with DAPI.

### Imaging and counting of cells

For retinas labeled with combined FluoroGold and DAPI, whole mounts or retinal sections were viewed using a Zeiss Axiophot fluorescent microscope (Carl Zeiss MicroImaging, Thornwood, NY). Images were captured using conventional photography on Ektochrome color 35 mm slide film (ISO 400, Eastman Kodak, Rochester, NY). Color slides were digitized using a Nikon Cool Scan scanner (Nikon, Melville, NY). Because digital images of color slide film often produce a blue hue to the image, all digital images were imported into Adobe Photoshop (Adobe Systems Inc., San Jose, CA) and processed to enhance the color differences in FluoroGold-labeled cells and nuclei stained with DAPI. All other samples were viewed using a Zeiss Axioplan 2 fluorescent microscope and captured as digital images using a black-and-white digital camera. Images were then pseudocolored using AxioVision 4.6.3.0 software (Carl Zeiss). Whole mounted retinas were all photographed using 400X magnification.

For counting of cells, digital images corresponding to different retinal regions were overlaid with random boxes 100 × 100 µm in size. This was designated as a single field. Fields imaged between 0 and 1,000 µm from the optic nerve head were considered part of the central retina, while those taken between 1,000 and 2,000 µm were considered to be from the mid-retina. The peripheral retina was considered as regions greater than 2,000 µm from the optic nerve head. All cells were classified as neurons. Ganglion cells were then counted within the boundaries of the box overlay, and the percentage of neurons represented by ganglion cells was calculated. Neurons were defined strictly from nuclear morphology using criteria described by others [1, 3, 15, 16]. Basically, a cell with a round or oval nucleus with minimal appearance of condensed heterochromatin and at least one prominent nucleolus was considered a neuron in the GCL.

## Results

### Historical values of ganglion cell percentage from the literature

Various methods have been used to identify and quantify ganglion cells in rats and mice. These methods and the resulting values are summarized in Table 1. Overall, various methods have yielded a large discrepancy in the percentage of neurons in the GCL that are ganglion cells. The estimate of 41.6% reported by Jeon and colleagues [1] represents one of the lowest values reported in the literature, yet this value has been the most commonly used for normalizing retinal ganglion cell loss.

### Ganglion cell percentages across the retina do not change

**Figure 1:**
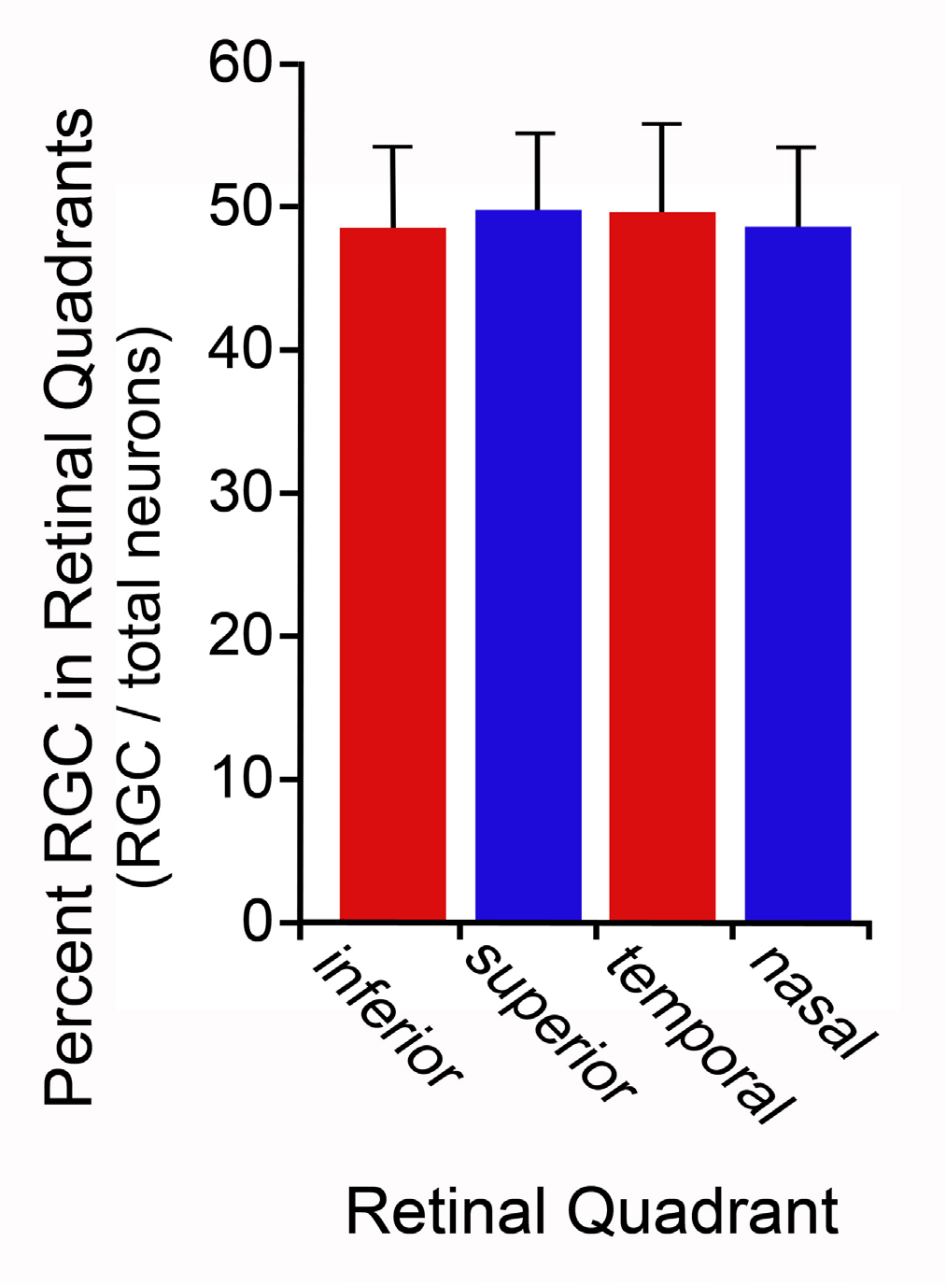
Histograph showing the percentage of ganglion cells that comprise the neurons in the ganglion cell layer. Ganglion cells were identified from retinas retrogradely labeled with FluoroGold and counterstained with TO-PRO-3. Data were collected from a total of 11 retinas (C57BL/6 mice). The percentage of ganglion cells did not differ among different retinal quadrants (ANOVA, p=0.798). Similarly, the percentage of neurons as ganglion cells did not vary significantly from the central to peripheral retina of each quadrant (p=0.09, data not shown).

In our initial experiments, we used retrograde labeling of FluoroGold to identify ganglion cells and TO-PRO-3 counterstaining to identify total neurons in the retinas of C57BL/6J mice (n=11 retinas). Data were collected as a function of the retinal quadrant, and secondarily stratified as central retina, mid-retina, or peripheral retina as detailed in the Methods section. An average of nine microscopic fields were analyzed for each quadrant (yielding a total of 99 fields/quadrant for analysis). In this study, the average ganglion cell percentage among all the retinas was 49.1±11.9%. This percentage did not vary among different quadrants (Figure 1; p=0.798, one-way ANOVA [ANOVA]) or from central to peripheral locations on the retina (p=0.09), even though overall total neuron and ganglion cell density varied across the mouse retina (p=0.002 and 0.031, respectively), consistent with other reports that ganglion cell density is highest in the nasal and inferior regions of the mouse retina [1, 15, 17].

### Ganglion cell percentages using different labeling methods

**Figure 2:**
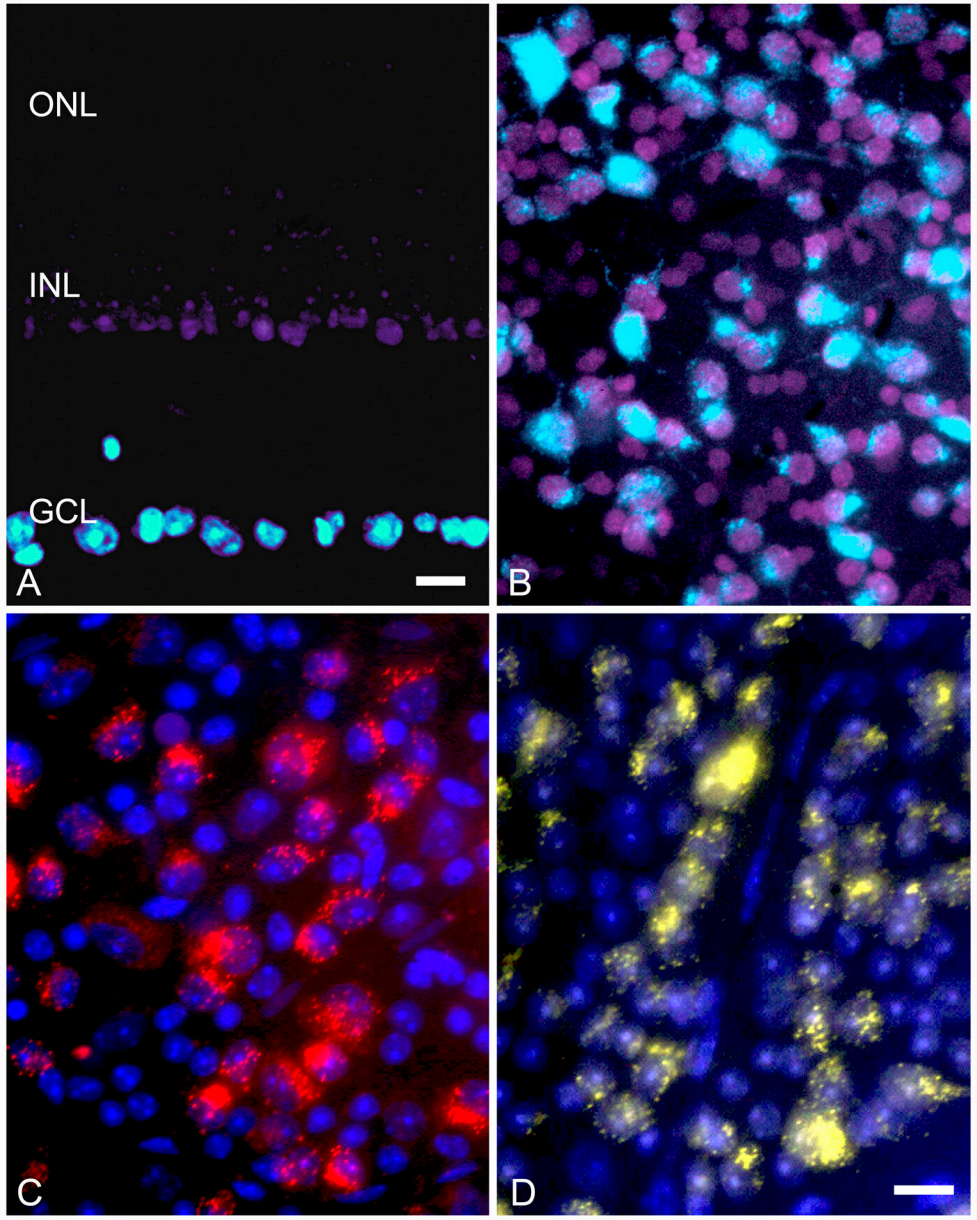
Images of mouse retinas with ganglion cells identified with retrograde labeling. **A** and **B**: A plastic section and whole mount of mouse (**A**) and rat (**B**) retinas, respectively, are labeled with a mixture of FluoroGold and 4',6-diamidino-2-phenylindole (DAPI, see Methods) stereotactically injected into the superior colliculus. The section in (**A**) shows that DAPI (purple) leaks from ganglion cells in the ganglion cell layer (GCL) and penetrates as far as the innermost layer of the inner nuclear layer (INL), while the outer nuclear layer (ONL) is unstained. FluoroGold (light blue) remains in the ganglion cells of the GCL. The whole mounted retina shows the distribution of FluoroGold/DAPI positive cells, relative to the cells stained with DAPI only. These images are electronically enhanced from digitized 35 mm color slide film. **C**: The whole mount of a retina is stained with retrograde 1,1'-dioctadecyl-3,3,3 3′-tetramethylindocarbocyanine perchlorate (DiI) label (red, DAPI counter stain). **D**: The whole mount of a retina is stained with retrograde hydroxystilbamidine (FluoroGold; yellow, TO-PRO-3 counterstain). In the latter two examples, the retrograde dyes were applied using gel-foam soaked pledgets placed over the exposed superior colliculus. Size bar in **A**=20 µm. Size bar in **B, C, D**=10 µm.

Table 2 shows the summary of metrics obtained from retinas in which ganglion cells were identified using different labeling methods. Figure 2 shows images obtained from retinas stained using different retrograde labeling paradigms. In the first image, FluoroGold and DAPI were used as the retrograde tracer. Both dyes entered the ganglion cells by way of axonal transport, but DAPI diffused across the plasma membranes of these cells and labeled other cells in the GCL. Within as few as 3 days after stereotactic injection, DAPI was identified as labeling the inner most neurons in the inner nuclear layer (Figure 2A). Rat (Figure 2B) and mouse retinas labeled with this method exhibited slightly more than 50% of the neurons as ganglion cells (Table 2). Retinas labeled retrogradely with either DiI or FluoroGold only, and then counterstained with DAPI or TO-PRO-3 after whole mounting (Figure 2C,D) exhibited a similar proportion of neurons as ganglion cells (49.1%; Table 2).

**Figure 3:**
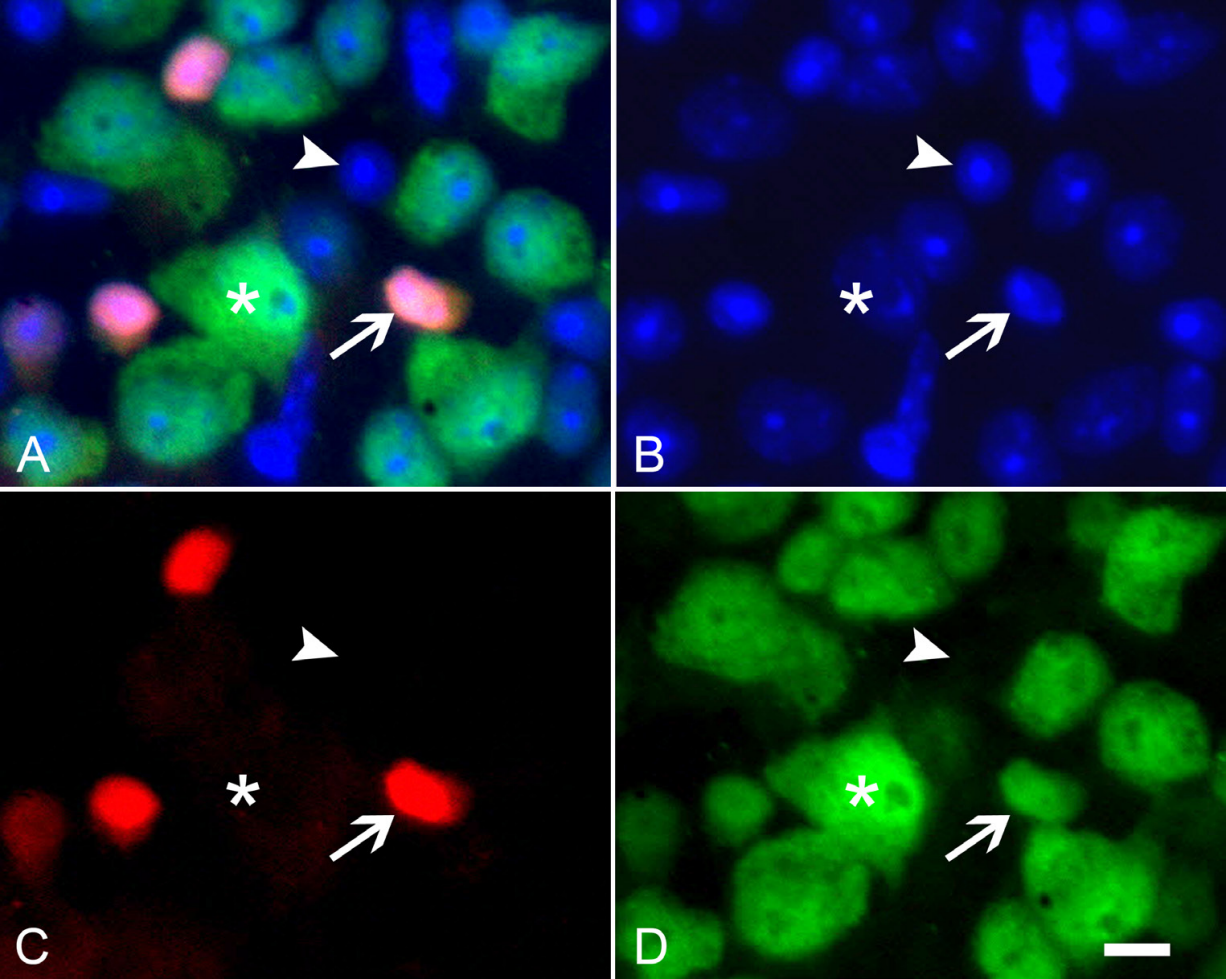
Immunofluorescent image of NeuN labeling of presumptive ganglion cells and cholinergic amacrine cells. **A**: The merged image of a region of a whole mounted retina from mice expressing tomato fluorescent protein in cholinergic amacrine cells (red, panel **C**), immunolabeled for neuronal-specific nuclear protein (NeuN; green, panel **D**), and counterstained with 4',6-diamidino-2-phenylindole (DAPI, blue, panel **B**) is shown. Some neuron-like cells do not label with NeuN (exemplar indicated by arrowhead). NeuN colocalizes with the label for cholinergic amacrines (exemplar indicated with an arrow), in addition to other cell types (asterisk). Size bar=10 µm.

In addition to retrograde labeling methods to identify ganglion cells, we also examined protocols that utilize specific immunostaining for reported ganglion cell markers. Several recent studies have adopted using NeuN immunostaining to identify ganglion cells [13, 18, 19]. Evaluation of the percentage of neurons expressing NeuN in the mouse retina yielded a relatively high proportion of cells (68.33±5.51%, Table 2) compared to the retrograde labeling paradigms. Others, however, have observed variable staining of amacrine neurons with NeuN [20]. Staining of mouse retinas with cholinergic amacrine cells identified with Tomato fluorescent protein indicated robust colocalization of NeuN immunoreactivity with these cells (Figure 3).

**Figure 4:**
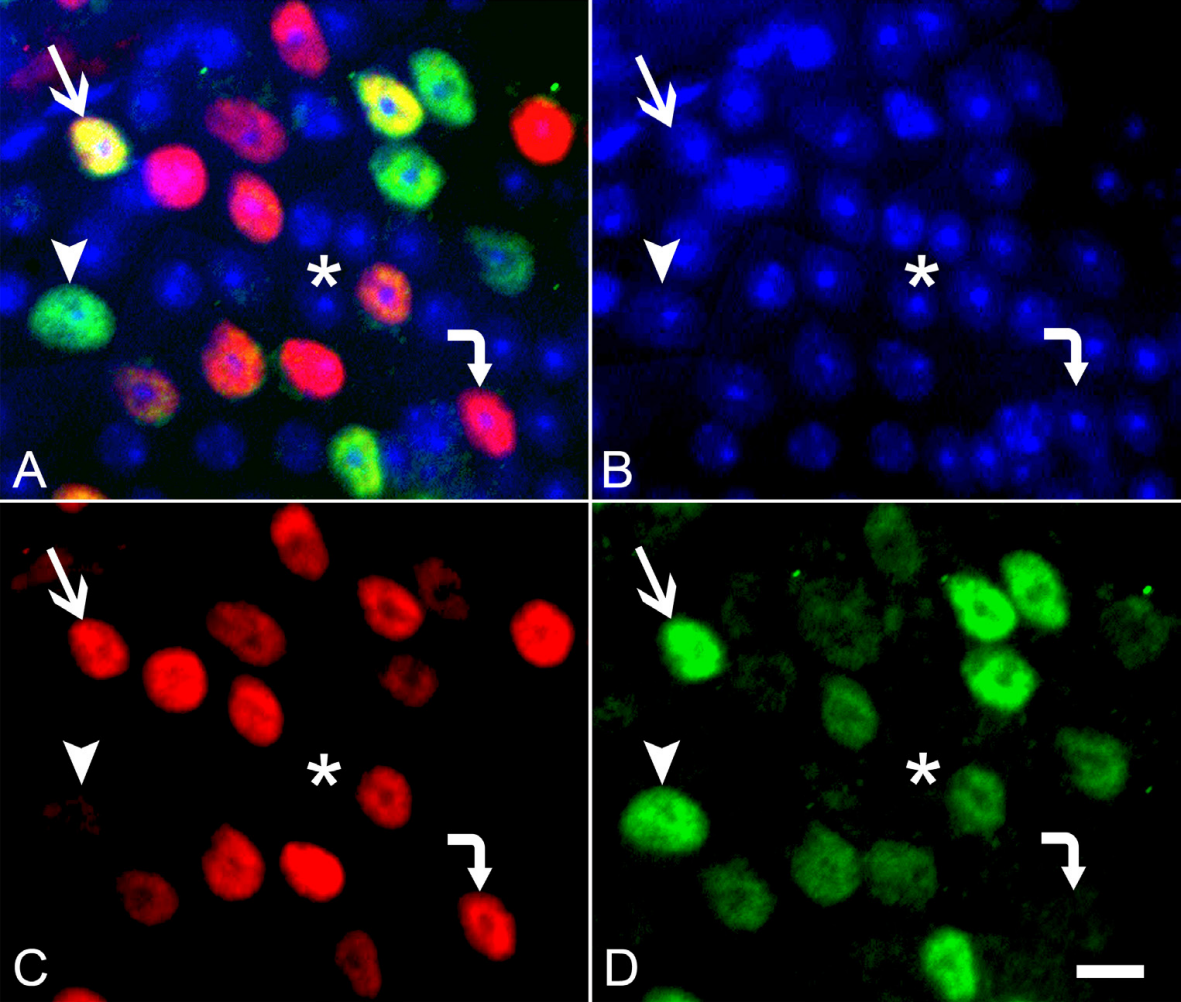
Immunofluorescent image of BRN3 positive ganglion cells. **A**: The merged image of BRN3A (red, panel **C**), BRN3B (green, panel **D**) and counterstained with 4',6-diamidino-2-phenylindole (DAPI, blue, panel **B**) is shown. This double labeling reveals four distinct classes of cell types. A cluster of cells that do not label with either BRN antibody are indicated with an asterisk. Most cells expressing BRN3 are positive for both proteins (example indicated with a straight arrow), while a minority express either BRN3A alone (example indicated with a bent arrow) or BRN3B alone (example indicated with an arrowhead). Size bar=10 µm.

Ganglion cells specifically express members of the class IV-POU domain transcription factors known as BRN3 [21, 22]. Several studies have used either BRN3A or BRN3B immunostaining to identify ganglion cells in models of optic nerve damage (see references [2, 23, 24] for some examples). Using BRN3A to identify ganglion cells, we estimate that 44.76±9.1% of the neurons express this marker, while an estimated 54.05±12.79% of the neurons express BRN3B. Some ganglion cells express both markers, while a smaller population express only one or the other (Figure 4). Combining all BRN3 positive cells in this calculation yielded an estimate of 61.33±13.9% of the neurons in the GCL were ganglion cells (Table 2).

## Discussion

Our survey of retrograde methods to label ganglion cells yielded an average of 51.25% of neurons in the GCL of the mouse retina that comprise this cell type. This is higher than the commonly used value of 41.6% reported by Jeon and colleagues [1], who used axon counts to determine ganglion cell number in C67BL/6 mice (44,860 based on axon counts from three optic nerves). Conversely, Williams and colleagues reported a value of 54,416±4,896 based on the axon counts from 21 nerves of C67BL/6 mice [25]. Since this latter value is based on a much greater sample size, this value may be a better estimate of axon number in this strain than values obtained by other groups. Using the Williams number to calculate the percentage of ganglion cells, divided by the Jeon estimate of total neurons, yielded a value of 49.4% for C57BL/6 mice. The same calculation using the Quigley estimate for total neurons [3] changed the percentage to 57.7% for the same strain (wild-type mice).

The use of ganglion cell specific markers to identify these cells tends to provide higher, more variable percentages compared to retrograde labeling. This could be a result of the selective expression of these proteins within subsets of ganglion cells, which may have resulted in the low estimates (36%–40%) obtained using SNCG immunolabeling [3] (see also Table 1). Interestingly, in situ hybridization of mouse retinal whole mounts, using *Sncg* antisense probes, yielded values more in line with retrograde labeling methods (45%–48%) [26]. Alternatively, different cell types in the GCL, in addition to ganglion cells, may label with the same marker. Previously, we had used *Thy1* mRNA in situ hybridization to identify ganglion cells, which produced estimates of ganglion cell percentages higher than retrograde methods (Table 1). These high estimates may reflect *Thy1* expression in Müller cells with stem-cell like properties [27].

Consistent with other reports that NeuN labels ganglion and amacrine cells [20], the percentage of neurons identified by this stain is also higher than conventional retrograde methods (about 68% versus about 51%). Double labeling for cholinergic amacrine cells and NeuN verified that at least this population of amacrines, reported to be 19.5% of the displaced amacrine cell population [1], was strongly positive for the NeuN antigen. Buckingham and colleagues [18] also recognized this fact and provided a correction factor (X 0.838) in their study to better estimate ganglion cell numbers in the retinas of aged DBA/2J mice.

BRN3 labeling of ganglion cells in the rat retina has been elegantly studied by Nadal-Nicolás and colleagues [14, 23], and provides an example of differential expression of markers within different populations of ganglion cells. BRN3A labels between 92.2% and 96.4% of FluoroGold-labeled ganglion cells (albino and pigmented strains, respectively). In both strains, approximately 65% of the BRN3A+ cells also express BRN3B, while only about 7% of the BRN3B+ cells do not express BRN3A [14]. These proportions of single- and double-labeled cells appear to be different in the mouse retina. An estimate of ganglion cell percentage is higher for BRN3B staining, compared to BRN3A (54.05% versus 44.76%), while the rat retina has a higher proportion of ganglion cells expressing BRN3A. Approximately 12% and 27% of the predicted ganglion cells in the mouse were individually positive for either BRN3A or BRN3B, compared to a predicted 35% and 11%, respectively, extrapolated from data presented by Nadal-Nicolás et al. [14]. Percentages of double-labeling in the mouse retina, similar to ours, were evident, however, in experiments using sparse conditional activation of different *Brn3* alleles linked to alkaline phosphatase expression [28]. In this study, 13% of A+ ganglion cells did not express BRN3B, while 22% of B+ cells did not express BRN3A. This implies that the expression of BRN3 family proteins may be different between species. Combining all BRN3 positive cell totals, we calculate that 61.33% of the neurons are ganglion cells in the mouse GCL using this labeling method.

A potential limitation to the data presented here is that nearly all the estimates of the percentage of retinal ganglion cells in the mouse retina were obtained from the C57BL/6 strain. There is clearly variation in the total numbers of ganglion cells between strains [25, 29], but there has been only limited evaluation of displaced amacrine cell variation among mouse strains. Whitney and colleagues [30] evaluated the numbers of dopaminergic amacrine cells across strains. A comparison of common strains between this study and that of Williams et al. [25] for ganglion cell numbers showed that the variation of both cell populations matched among the strains (DBA/2J > C57BL/6 > A/J). Consistent with this finding, Li et al. [16] showed that the percentage of ganglion cells for DBA/2J and BALB/cByJ was remarkably similar. Overall, although caution should be used to simply superimpose a value derived from one strain to all others, these data suggest that the proportion of amacrine and ganglion cells remains relatively fixed between strains, even though the total numbers of neurons vary.

In summary, the methods for identifying ganglion cells in the mouse retina clearly have variable factors associated. Due to the relative consistency we obtained using different retrograde dyes, we predict that these methods provide a more accurate estimate of ganglion cell number over methods using ganglion cell specific markers. Several important experiments are being conducted to assess the cellular and molecular pathways associated with ganglion cell loss after optic nerve damage. Various methods, for several reasons, utilize total neuronal cell counts in the GCL as the outcome metric for analysis. We propose that studies using a correction factor to better estimate the presence or absence of ganglion cells from this outcome metric should adopt approximately 50% as the proportion of retinal ganglion cells that comprise the total neuronal population in the GCL of the rat and mouse retina. Overall, however, methods of quantifying ganglion cells should be appropriate for the design of the experiments.

## 

**Table 1:** Historical summary of ganglion cell percentages

Study	Method	Total Neurons	Total RGCs	Percentage	Species (strain)
Drager and Olsen [15]	Retrograde Label^a^	117,000	70,000	59.8%	*M. musculus* (C57BL/6)
Jeon et al. [1]	Axon Counting^b^	110,242±5826	44,860±3125	41.6%	*M. musculus* (C57BL/6)
Li et al. [31]	*Thy1* in situ^c^	N/A	N/A	67.5±6.5%	*M. musculus* (CB6F1)
Schlamp et al. [9]	*Thy1* in situ^d^	N/A	N/A	68.1±5.3% 57.5±13.1%	*R. norvegicus* (Brown Norway)
Li et al. [16]	Axon Counting^e^	118,416±12,313 (DBA/2J) 90,788±11,200 (BALB/cByJ)	72,175±17,554 (DBA/2J) 54,740±8,484 (BALB/cByJ)	61.0% (DBA/2J) 60.3% (BALB/cByJ)	*M. musculus*
Pang and Wu [32]	Retrograde Label^lf^	113,222±4,430	49,823±1,792	44%	*M. musculus* (C57BL/6)
Quigley et al. [3]	SNCG immunolabel^g^	N/A	N/A	40.3±5.9% (WT) 36.1±5.2% (*Jnk*^−/−^)	*M. musculus* (C57BL/6)
Quigley et al. [3]	Axon Counting^h^	94,229	46,807±4,785	49.7%	*M. musculus* (C57BL/6)

The summary table shows a chronological order only of studies that report ganglion cell number as a percentage of neurons in the GCL. The Method column refers to the general method used to identify retinal ganglion cells in either the mouse of rat retina. Details of the methods used in each study are: ^a^Total non-vascular cells were counted from Nissl-stained retinal whole mounts. RGCs were identified by Horse Radish Peroxidase (HRP) retrograde labeling, where the optic track on one side was macerated at the level of the Lateral Geniculate Nucleus and 1 mg of HRP was applied to the lesion. A total of 12 mice were examined (12 retinas with HRP and the contralateral eye for Nissl). Of note, 69% of the cells with Nissl-substance were also HRP labeled. The non-HRP Nissl+ labeled cells were on average 21% smaller in area. Sample sizes were estimated at 6.1% of the retina. The source of the mice was not given. ^b^Total neurons were counted in 9 retinal whole mounts by labeling nuclei with an ethidium homodimer. Neurons were classified by morphological criteria of the nuclear stain (round to oval nucleus and prominent nucleoli). Ganglion cell numbers were estimated from axon counts of 3 optic nerves, which were evaluated by transmission electron miccroscopy (osmium tetroxide post-fix and uranyl acetate stain). The source of mice was Charles River. ^c^Control mouse retinas were subjected to in situ hybridization for *Thy1* mRNA using a block tissue staining protocol. After staining, the retina blocks were embedded in JB-4 Plus plastic and sectioned. Sections were counter stained with DAPI to identify nuclei. A total of 8 control retinas were examined, with 12–15 sections per retina being analyzed (cells counted along a 400 µm length per section). Of note, after optic nerve crush, these studies noted a loss of 57.8±8.1% of the neurons in the GCL after 3 weeks post optic nerve crush. Total neurons and RGCs were not estimated. The source of mice was an inbred colony of the investigator. ^d^Same in situ hybridization protocol as above (c) except that eyes were from rats with ocular hypertension. Control eyes (n=4) had the higher percentage, while eyes with minimal damage (based on semiquantitative evaluation of paraphenaline diamine-stained sections of optic nerves, n=5) had the lower percentage. Data was not significantly different. The source of rats was not given. ^e^Total neurons were counted from Nissl-stained retinal whole mounts prepared from ~30 mice each individual strain (~10% retinal area surveyed per retina). Ganglion cell numbers were estimated from silver impregnated sections of 3 sections each of 5 separate nerves from each individual strain (9 1000×fields per nerve). The source of mice was Jackson Laboratories. ^f^Cells in the GCL were double labeled using a retrograde approach by dipping the optic nerve stump of enucleated eyes into a mixture of Lucifer Yellow (LY, stays in ganglion cells) and neurobiotin (NB, leaks out of ganglion cells). LY and NB double-labeled cells were considered ganglion cells. TO-PRO-3 was used as a nuclear counter stain. Sample size estimated at ~10% of the retina and a total of 9 mice were analyzed. The source of mice was Jackson Laboratories. ^g^In the first method reported in this study, total neurons (per field) were identified in 4 retinal whole mounts based on nuclear morphology after DAPI counter stain. Ganglion cells were identified by immunostaining the wholemount for SNCG protein using a commercial antibody. No indication was given of the numbers of fields counted for the immunostained samples. The total neurons and retinal ganglion cells were not estimated using this method. ^h^In the second method reported in this study, values for total neurons and total retinal ganglion cells were extrapolated from data presented for wild-type mice in this report. They were not used to calculate the percentage of ganglion cells by the authors. Total ganglion cell numbers were estimated from osmium and toluidine blue stained sections of 10 optic nerves. Total neurons were extrapolated from DAPI-stained whole mounts of wild-type mice (Table 1, 6596 neurons were counted, which was estimated as 7% of the total retina).

**Table 2:** Summary of ganglion cell percentages with different labeling paradigms

Method	Number Retinas Analyzed	Total Fields Counted	RGC Percentage (mean ± SD)	Range
FG_r_-DAPI_r_	6 (3 mice)	218	53.51±17.10%	94% to 20%
FG_r_-DAPI_r_	2 (2 rats)	70	50.21±12.66%	84% to 23%
FG_r_-TO-PRO-3_cs_	11 (7 mice)	138	49.10±11.88%	78% to 20%
DiI_r_-DAPI_cs_	6 (6 mice)	57	51.14±9.68%	71% to 33%
NeuN-DAPI_cs_	4 (2 mice)	16	68.33±5.51%	80% to 62%
BRN3A-DAPI_cs_	4 (2 mice)	30	44.76±9.13%	65% to 29%
BRN3B-DAPI_cs_	4 (2 mice)	30	54.05±12.79%	79% to 31%
BRN3 combined	4 (2 mice)	30	61.33±13.9%	87% to 37%

The method column refers to the methods use to identify both retinal ganglion cells and the nuclear stain for estimating total neurons. Retrograde dyes are indicated with a subscript “r,” while counter-stains are represented with a subscript “cs.” The data designated as “BRN3 combined” represents all cells that were positive for BRN3A + BRN3B in the same field. Approximately 61% of these cells were positive for both BRN3 proteins. A single field represents an area of 10^4^ µm^2^, and contained approximately 100 neurons.
